# Preliminary Studies to Characterize the Temporal Variation of Micronutrient Composition of the Above Ground Organs of Maize and Correlated Uptake Rates

**DOI:** 10.3389/fpls.2017.01482

**Published:** 2017-09-01

**Authors:** Karla Vilaça Martins, Durval Dourado-Neto, Klaus Reichardt, Quirijn de Jong van Lier, José Laércio Favarin, Felipe Fadel Sartori, Guilherme Felisberto, Simone da Costa Mello

**Affiliations:** ^1^Crop Science Department, Escola Superior de Agricultura “Luiz de Queiroz” (ESALQ), Universidade de São Paulo Piracicaba, Brazil; ^2^Center for Nuclear Energy in Agriculture, University of São Paulo Piracicaba, Brazil

**Keywords:** *Zea mays*, micronutrient content, micronutrient partition, productivity, agricultural modeling

## Abstract

The improvement of agronomic practices and the use of high technology in field crops contributes for significant increases in maize productivity, and may have altered the dynamics of nutrient uptake and partition by the plant. Official recommendations for fertilizer applications to the maize crop in Brazil and in many countries are based on critical soil nutrient contents and are relatively outdated. Since the factors that interact in an agricultural production system are dynamic, mathematical modeling of the growth process turns out to be an appropriate tool for these studies. Agricultural modeling can expand our knowledge about the interactions prevailing in the soil-plant-atmosphere system. The objective of this study is to propose a methodology for characterizing the micronutrient composition of different organs and their extraction, and export during maize crop development, based on modeling nutrient uptake, crop potential evapotranspiration and micronutrient partitioning in the plant, considering the production environment. This preliminary characterization study (experimental growth analysis) considers the temporal variation of the micronutrient uptake rate in the aboveground organs, which defines crop needs and the critical nutrient content of the soil solution. The methodology allowed verifying that, initially, the highest fraction of dry matter, among aboveground organs, was assigned to the leaves. After the R_1_ growth stage, the largest part of dry matter was partitioned to the stalk, which in this growth stage is the main storage organ of the maize plant. During the reproductive phase, the highest fraction of dry matter was conferred to the reproductive organs, due to the high demand for carbohydrates for grain filling. The micronutrient (B, Cu, Fe, Mn, and Zn) content follows a power model, with higher values for the initial growth stages of development and leveling off to minimum values at the R_6_ growth stage. The proposed model allows to verify that fertilizer recommendations should be related to the temporal variability of micronutrient absorption rates, in contrast to the classic recommendation based on the critical soil micronutrient content. The maximum micronutrient absorption rates occur between the reproductive R_4_ and R_5_ growth stages. These evaluations allowed to predict the maximum micronutrient requirements, considered equal to respective stalk sap concentrations.

## Introduction

Maize (*Zea mays* L.) is the most cultivated cereal worldwide, mainly because of the different ways of consumption, as human food and animal feed, as well as many byproducts for the high technology industry (Edwards, 2009). Brazil is the world's third largest maize producer, behind the United States and China. In Brazil, maize is general grown in two cropping seasons, and covered an area of 15,627,300 ha in the 2014/2015 season. The national average yield triplicated from 1,632 kg ha^−1^ to 5,382 kg ha^−1^ over the last 40 years (CONAB, 2015). Such increases in productivity are due to the development of agriculture in relation to the breeding of plants and management practices, including the correction and fertilization of soils (Bender et al., 2013; Ciampitti et al., 2013). However, the application of micronutrients also played an important role, although the information on their absorption and partition by the maize plant rely on older literature as stated by Ciampitti et al. (2013). The most recent studies on the subject have been carried out mainly in the United States. Few studies were performed on the absorption and nutrient partitioning in modern maize hybrids used in Brazil (Von Pinho et al., 2009). The Brazilian lime and fertilizer recommendations are mainly based on 15–20-year-old studies such as van Raij et al. (1996), Ribeiro et al. (1999), Oliveira (2003), SBCS (2004) and Cantarutti et al. (2007).

In addition, agricultural production systems have also changed, with higher plant densities, reduced seed spacing, use of agrochemicals for crop protection and use of transgenic hybrids (Bender et al., 2013).

The use of increasingly growing high-tech crops may have changed the dynamics of absorption and partition of nutrients by the maize crop. Therefore, studies on the current absorption patterns and partition of micronutrients are welcome to update official fertilizer recommendations, which are still based on soil chemical analyses. This is essentially a static approach, whereas processes during crop development are dynamic. For example, using only soil chemical analyses does not allow to consider variations of the critical micronutrient content among phenological growth stages, expected productivity and soil and climatic interactions.

The proposed model will be useful for the improvement of the traditional fertilizer methodology based on soil analysis, giving emphasis to the plant as a nutrient extractor. The model considers that the fertilizer recommendation should be based on the temporal variability of the nutrient absorption rate, in comparison to the classic recommendation based on the critical soil nutrient content.

This study is based on the following hypotheses: (i) the maximum micronutrient concentration in the sap depends on productivity and transpiration, (ii) the micronutrient content in the different organs is characterized by a power function and does not depend on productivity, and (iii) the nutrient with concentration in the sap equal to the required critical concentration of the crop, limits productivity (Liebig Law).

This preliminary study aims to propose a methodology for characterizing the composition of different organs and extraction, distribution and export of the micronutrients boron (B), copper (Cu), iron (Fe), manganese (Mn), and zinc (Zn) during maize plant development. Based on modeling micronutrient uptake, crop potential evapotranspiration and micronutrient partition in the plant, taking into account the micronutrient uptake rate in a given production system, it contributes to an improvement in the recommendation of these micronutrients.

## Materials and methods

### Environmental conditions

A field experiment with maize was carried out in Piracicaba, state of São Paulo, Brazil (22° 41′ S; 47° 38′ W, 546 m above sea level) to characterize the temporal variation of above ground dry matter accumulation and micronutrient contents from sowing until physiologic maturity.

The climate is of the Köppen Cwa type (Alvares et al., 2013), with a rainy summer and dry winter, annual average air temperature 21.4°C and yearly rainfall 1,257 mm. The reference evapotranspiration (ETo, mm d^−1^) was calculated by the Penman-Monteith method (Allen et al., 1998), and the water balance was established according to Thornthwaite and Mather (1955).

The soil was classified as a typical Hapludox as defined by the USDA Soil Taxonomy (Soil Survey Staff, 1975). The micronutrients B (determined in hot-water-soluble method developed by Berger and Truog, 1940), Cu, Fe, Mn and Zn (determined in DTPA pH 7.3 method developed by Lindsay and Norvell, 1978) concentrations in the soil were, respectively, 0.32, 4.0, 15.0, 16.8, and 2.5 mg dm^−3^.

### Cropping system characterization

Maize was sown on 26 March 2013, using a population of 65,000 plants ha^−1^ (spacing between rows of 0.45 m). The maize simple hybrid DKB 390 VT PRO™ 2 was chosen due to its favorable features, specifically: (i) high potential productivity, (ii) *YieldGard* technology (tolerance to *Spodoptera frugiperda, Helicoverpa zea*, and *Diatraea saccharalis*) and (iii) RR technology (tolerance to the glyphosate herbicide).

For dry matter composition characterization, a homogeneous single plot of 5,000 m^2^ was sown and managed in the same way applying nitrogen (30 kg ha^−1^ of N), phosphorus (80 kg ha^−1^ of P_2_O_5_) and potassium (40 kg ha^−1^ of K_2_O). An additional of 90 kg ha^−1^ of N was applied at the V_4_ phenological stage.

The seed treatment consisted of insecticide and fungicide applications (Fipronil, Pyraclostrobin and Thiophanate-methyl at a rate of 200 mL per 100 kg of seeds).

### Sampling description

The plot was subdivided into 315 parcels used for sampling of the above ground plant parts. Each parcel of 12.6 m^2^ consisted of four maize lines 7 m long, the central ones used for plant sampling. With this large number of parcels, it was possible to randomly sample only two plants per parcel during the complete cycle of the crop.

Samplings consisted of plant collection at times according to the growth stages defined by Ritchie et al. (1996), as follows: V_2_, V_4_, V_6_, V_8_, and V_10_, which occurred at 14, 21, 28, 35, and 42 days after seeding (*t, d*), respectively (Table 1). Sixty plants were collected at each sampling date, two per plot, one of each central line, using 30 parcels chosen randomly over the whole plot. The 60 sampled plants were subdivided, also randomly, into six replicates (composed samples) of 10 plants each for dry matter and chemical analyses.

**Table 1:** Description of sampling (S) date (D), growth stages (GS), accumulated degree-days (DD, ^o^Cd) and relative development (R_d_, %) based on DD of the maize crop (hybrid DKB 390 VT PRO 2), during vegetative and reproductive phases, as a function of time (t, d), from March 26 to August 12, 2013.

**S**	**Vegetative phase**					**S**	**Reproductive phase**				
	**t**	**D**	**GS^a^**	**DD^b^**	**R_d_**		**t**	**D**	**GS^a^**	**DD^b^**	**R_d_**
–	0	March 26	Seeding	–	–	8	70	June 4	R_1_	863	54.1
–	7	April 2	V_E_	0	0.0	9	77	June 11	R_2_	943	59.1
1	14	April 9	V_2_	215	13.5	10	84	June 18	R_2_/R_3_	1,021	64.0
2	21	April 16	V_4_	307	19.2	11	91	June 25	R_3_	1,097	68.7
3	28	April 23	V_6_	385	24.1	12	104	July 8	R_4_	1,249	78.3
4	35	April 30	V_8_	474	29.7	13	111	July 15	R_4_/R_5_	1,325	83.0
5	42	May 7	V_10_	567	35.5	14	118	July 22	R_5_	1,411	88.4
6	50	May 15	V_12_	653	40.9	15	127	July 31	R_5_/R_6_	1,462	91.6
7	56	May 21	V_15_	728	45.6	16	139	August 12	R_6_	1,596	100.0

a *Ritchie et al. (1996)*.

b *DD: 10°C as the lower base temperature and 35°C as the upper base temperature*.

At V_12_, V_15_, R_1_, R_2_, R_2_, R_3_, R_4_, R_4_, R_5_, R_5_, and R_6_, which occurred at 50, 56, 70, 77, 84, 91, 104, 111, 118, 127, and 139 days, respectively (Table 1), the number of harvested plants was reduced to 30 plants per sampling. Therefore, the resulting composed samples consisted of five plants.

In this way, during the experimental period, 630 plants were sampled, corresponding to 1.93% of all plants. To determine the crop development growth stage, phenological characterization was performed every 2 days during the crop cycle, according to Ritchie et al. (1996).

Plant organ samples were dried at 65°C to characterize leaf, stalk, tassel, ear, straw, style-stigma and total dry matter. Subsamples were used for micronutrient analyses.

The leaf area was evaluated with a LI-COR® sensor (model Li-3100C, Lincoln, Nebraska, USA) allowing leaf area index (LAI, m^2^ m^−2^) estimation during crop development. LAI was determined in all growth stages, with six replicates, using the same leaves for dry matter and chemical analyses.

Harvest was performed at physiologic maturity (R_6_ growth stage), collecting all plants of the central two lines of 7 m, discarding 0.5 m at each border. Grain yield was estimated from the weight of 1,000 seeds at 13% water content.

### Basic hypothesis of the micronutrient absorption model

Considering that at a given time t (*d*) within the crop cycle, plants have accumulated a mass of dry matter per area *D* (kg ha^−1^), with a nutrient content *N* (mg kg^−1^), the cumulative nutrient absorption *A* (kg ha^−1^) is given by the product of *D* and *N*.

Using observed data for parameterization, *D* (sigmoid function) and *N* (power function) were modeled as a function of time (*t*) using the following empirical equations:

(1) $$\begin{array}{rcl} D & = & k_{1}+\frac{k_{2}{k_{4}}^{2}}{{k_{4}}^{2}+{\left(t-k_{3}\right)}^{2}} \end{array}$$

(2) $$\begin{array}{rcl} N & = & k_{5}.t^{k_{6}} \end{array}$$

in which *k*_1_ (kg ha^−1^), *k*_2_ (kg ha^−1^), *k*_3_ (d), *k*_4_ (d), *k*_5_ (mg kg^−1^ *d* ^−*k*_6_^) and *k*_6_ are empirical fitting parameters calibrated from experimental data of *D* and *N* by minimizing the sum of square errors.

The development of the general model is based on the growth curve of the maize plant given by the accumulation of the total dry matter, the sigmoidal Equation (1). For the temporal changes of the micronutrient content in the plant, the power function 2 was chosen.

Based on the dry matter production curve *D* and micronutrient content *N* of the above ground plant, a model was proposed to characterize the nutrient absorption *A*:

(3) $$\begin{array}{r} A=D.N=k_{1}k_{5}.t^{k_{6}}+\frac{k_{2}{k_{4}}^{2}k_{5}.t^{k_{6}}}{{k_{4}}^{2}+{\left(t-k_{3}\right)}^{2}} \end{array}$$

The shape of the sigmoidal curve represents a positive increase in dry matter accumulation with increasing rates in the vegetative growth stages and with decreasing rates in the reproductive growth stages.

### Application of the proposed model

**Figure 1:**
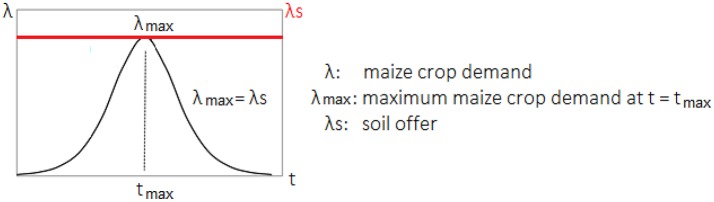
Micronutrient absorption rate (λ, mgha^−1^d^−1^) as a function of time (t, d), for the determination of the maximum absorption rate (λ_max_, mgha^−1^d^−1^) corresponding to the rate of the soil supplying capacity (λs, mgha^−1^d^−1^), considered sufficient during the crop cycle for a given productivity.

To calibrate the absorption rate curve (λ, mg ha^−1^ d^−1^) (Figure 1) for each of the micronutrients, the following equation was used:

(4) $$\begin{array}{l} \lambda =\frac{dA}{dt}=k_{1}k_{5}.k_{6}t^{k_{6}-1}\\ \;+k_{2}{k_{4}}^{2}k_{5}\left\{\frac{k_{6}t^{k_{6}-1}}{\left[{k_{4}}^{2}+{\left(t-k_{3}\right)}^{2}\right]}-\frac{2t^{k_{6}}\left(t-k_{3}\right)}{{\left[{k_{4}}^{2}+{\left(t-k_{3}\right)}^{2}\right]}^{2}}\right\} \end{array}$$

Starting from the first derivative of the absorption rate of a micronutrient λ (or the second derivative of the absorption march – *A*, Equation 3):

(5) $$\begin{array}{r} f\left(t\right)=\frac{d\lambda }{dt}=\frac{d^{2}A}{dt^{2}} \end{array}$$

The maximum micronutrient absorption rate (λ_max_) can be determined according to *f*(*t*) = 0. It is assumed to be related to the critical micronutrient content in the soil solution. The maximum absorption rate λ_max_ (maximum maize crop demand at t = t_max_ = t_i+1_) can be found at time t, in the iteration “i+1” (Figure 1), minimizing the sum of square errors, corresponding to d^2^A/dt^2^ = 0 using the iterative Newton-Raphson method:

(6) $$\begin{array}{rcl} t_{i+1} & = & t_{i}-\frac{f\left(t_{i}\right)}{f^{\prime }\left(t_{i}\right)} \end{array}$$

(7) $$\begin{array}{l} f\left(t\right)=C_{1}t^{k_{6}-2}+C_{2}t^{k_{6}-2}\left\{\frac{k_{6}}{T_{2}}-\frac{2t^{2}T_{1}}{{T_{2}}^{2}}\right\}\\ \;+\frac{C_{2}}{\left(k_{6}-1\right)}\left\{\frac{\left(-2k_{6}-4t\right)T_{1}-2t^{2}}{{T_{2}}^{2}}-\frac{8t^{2}{T_{2}}^{2}}{{T_{2}}^{3}}\right\}t^{k_{6}-1} \end{array}$$

where C_1_ [*C*_1_ = *k*_1_*k*_5_*k*_6_(*k*_6_ − 1)], C_2_ [$C_{2}=k_{2}{k_{4}}^{2}k_{5}\left(k_{6}-1\right)$], T_1_ [*T*_1_ = *t* − *k*_3_] and T_2_ [$T_{2}={k_{4}}^{2}+{\left(t-k_{3}\right)}^{2}$] are auxiliary variables.

(8) $$\begin{array}{l} f^{\prime }(t)=C_{1}\left(k_{6}-2\right)t^{k_{6}-3}+\;C_{2}\left(k_{6}-2\right)t^{k_{6}-3}\left(\begin{array}{l} \frac{k_{6}}{T_{2}}-\\ \frac{2t^{2}T_{1}}{{T_{2}}^{2}} \end{array}\right)\\ \;+\;C_{2}t^{k_{6}-2}\left\{\begin{array}{l} \frac{-2k_{6}T_{1}}{{T_{2}}^{2}}-\\ \frac{\left(4tT_{1}\;+\;2t^{2}\right){T_{2}}^{2}\;-\;8t^{2}{T_{1}}^{2}T_{2}}{{T_{2}}^{4}} \end{array}\right\}\\ \;+\;C_{2}t^{k_{6}-2}\left\{\begin{array}{l} \frac{\left(-2k_{6}\;-\;4t\right)T_{1}-2t^{2}}{{T_{2}}^{2}}-\\ \frac{8t^{2}{T_{1}}^{2}}{{T_{2}}^{3}} \end{array}\right\}\\ \;+\;\frac{C_{2}}{\left(k_{6}-1\right)}t^{k_{6}-1}\left\{\begin{array}{l} \frac{\begin{array}{l} 2\left[-2T_{1}-k_{6}-2t-t^{2}\right]T_{2}\;+\\ 8\left(k_{6}+2t\right){T_{1}}^{2}+8t^{2}T_{1} \end{array}}{{T_{2_{i}}}^{3}}-\\ \frac{16\left[t{T_{1}}^{2}\;+\;t^{2}T_{1}\right]T_{2}\;-\;48t^{2}{T_{1}}^{3}}{{T_{2}}^{4}} \end{array}\right\} \end{array}$$

If |*f*(*t*_*i*+1_)| < ε then *t*_max_ = *t*_*i*+1_, where ε is the maximum allowed error (10^−7^). The maximum absorption rate λ_max_ corresponds to the soil supplying capacity (λ*s*, mg ha^−1^ d^−1^) that meets the micronutrient demand during the entire crop cycle (Figure 1), so that:

(9) $$\begin{array}{l} \lambda_{\max}=\lambda \left(t_{\max}\right)=\lambda s=k_{1}k_{5}.k_{6}{t_{\max}}^{k_{6}-1}\\ \;+\;k_{2}{k_{4}}^{2}k_{5}\left\{\frac{k_{6}{t_{\max}}^{k_{6}-1}}{{k_{4}}^{2}+{\left(t_{\max}-k_{3}\right)}^{2}}-\frac{2{t_{\max}}^{k_{6}}\left(t_{\max}-k_{3}\right)}{{\left[{k_{4}}^{2}+{\left(t_{\max}-k_{3}\right)}^{2}\right]}^{2}}\right\} \end{array}$$

To estimate the maximum concentration Cc of a micronutrient in the maize stalk sap at time t (t = t_i+1_) of λ_max_ (when λ = λs), first the water flow rate absorbed by the roots is taken equal to the sum of the actual transpiration (*Ta*, mm d^−1^) and the absorbed water needed for daily plant growth (α, mm d^−1^).

Since micronutrient flux, here considered as λ_max_ for each micronutrient, is the product of the water flux and the maximum sap concentration (Cc, mg L^−1^), we have:

(10) $$\begin{array}{r} Cc=\frac{\lambda_{\max}}{10^{4}\left(Ta+\alpha \right)} \end{array}$$

where α is the daily absorbed water by the plant to form dry matter and the daily water retained by the plant (kg m^−2^ d^−1^). The maximum sap concentration (Cc*) can also be expressed in mmol L^−1^ dividing the term λ_max_/[10^4^(*Ta*+α)] by *M*, where *M* is the molecular mass (g mol^−1^) of the considered micronutrient (B, Cu, Fe, Mn, and Zn).

### Statistical analyses

All statistical analyses (regressions, model fitting) in this study were performed using the software *Table Curve 2D*, version 5.01 (Systat Software, 2000).

## Results

### Field experiment

**Figure 2:**
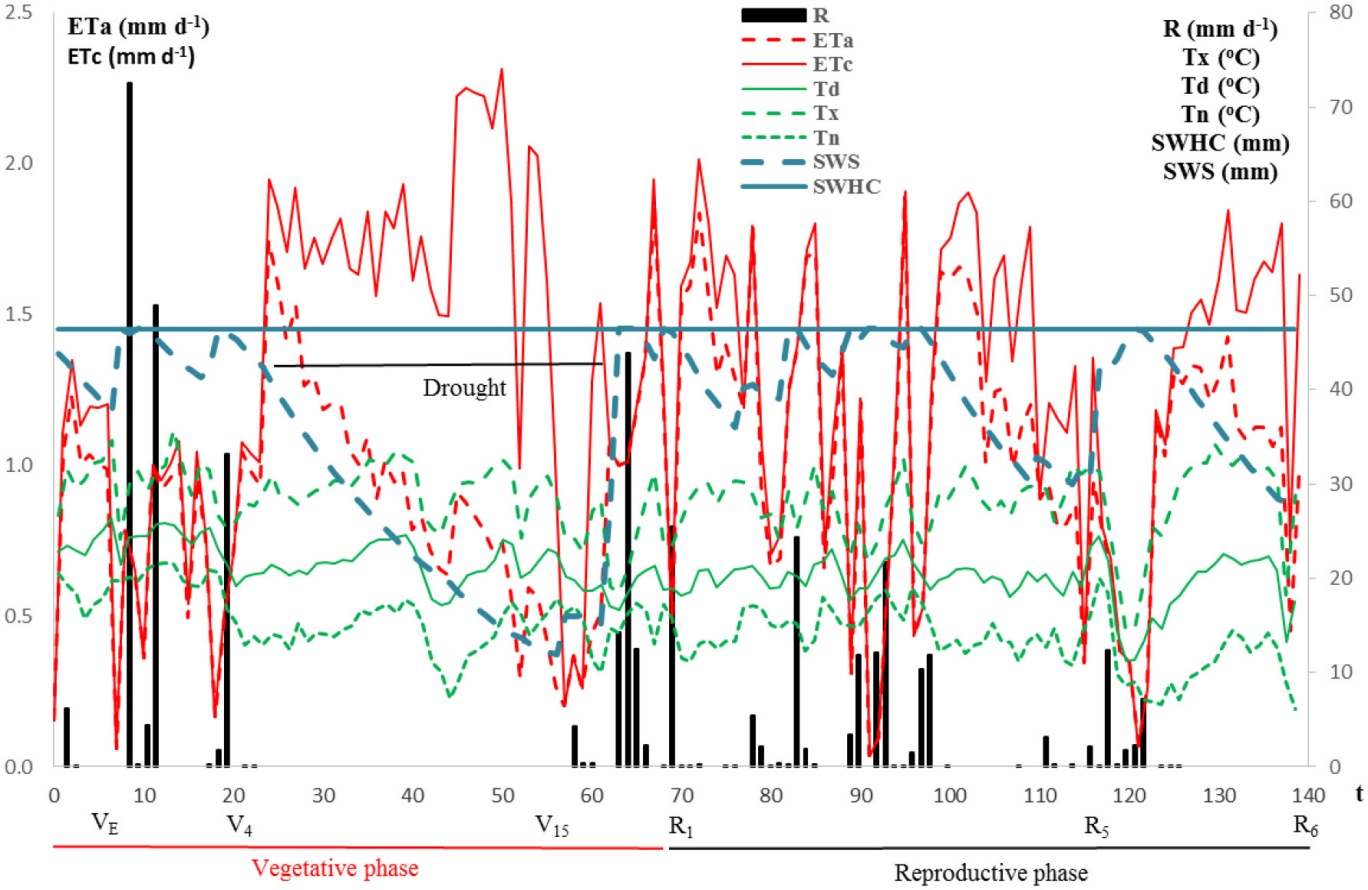
Minimum (*Tn*), average (*Td*) and maximum (*Tx*) air temperatures (°C), rainfall (R, mm d^−1^), potential (*ETc*, mm d^−1^), and actual (*ETa*, mm d^−1^) evapotranspiration, soil water holding capacity (SWHC, mm), and soil water storage (SWS, mm) as a function of time (t, d), from March 26 to August 12, 2013 (Time zero corresponds to sowing).

From March 26 to August 12, 2013, total precipitation was 409.6 mm (Figure 2). From seeding until the growth stage V_4_, there was no significant drought and from V_4_ to V_15_ growth stages (20–56 days) a drought period occurred. The grain productivity of the maize crop was 10,335 kg ha^−1^ (13% of seed water content).

### Climatic conditions and soil water storage

During the crop cycle, minimum (Tn), average (Td), and maximum (Tx) air temperatures oscillated, respectively, between 6.1 and 21.7°C (mean value: 14.6°C), 11.2 and 26.3°C (mean value: 20.7°C) and 14.6 and 35.7°C (mean value: 28.4°C). The actual evapotranspiration (ETa) varied between 0.1 and 1.9 mm d^−1^ most of the time (mean value: 0.96 mm d^−1^) (Figure 2).

During the dry spell, soil water storage reduced from 46.4 mm (soil water holding capacity—SWHC) to about 12.0 mm (Figure 2). From visual field observations, it was concluded that the crop water stress was not severe. Between 57 and 121 days, water supply by rain allowed a normal development of the crop (V_15_ to R_5_ growth stage, Figure 2). After 121 days, there was no more rain until maturity (R_6_ growth stage) and the harvest could be performed under excellent conditions.

### Leaf area index and dry matter accumulation

Positive increments of dry matter were observed from the onset of growth and development until the end of the vegetative phase (R_1_ growth stage) (Table 1), when the total dry matter reached 7.3·10^3^ kg ha^−1^ (Table 2).

**Table 2:** Average values of leaf area index (LAI, m^2^m^−2^) and dry matter (kgha^−1^) of leaf, stalk, tassel, ear, style-stigma, straw and total of the maize crop (hybrid DKB 390 VT PRO 2) as a function of time (t, d).

**t**	**LAI**	**Dry matter (kg ha**^**−1**^**)**						
		**Leaf**	**Stalk**	**Tassel**	**Ear**	**Style-stigma**	**Straw**	**Total**
14	0.06	18.2	7.8	.	.	.	.	26.0
21	0.28	100.1	59.2	.	.	.	.	159.3
28	0.75	286.0	203.5	.	.	.	.	489.5
35	1.56	655.2	1,007.5	.	.	.	.	1,662.7
42	2.47	1,302.6	1,106.3	.	.	.	.	2,408.9
50	3.35	2,031.9	2,779.4	.	.	.	.	4,811.3
56	3.93	2,782.0	3,773.3	.	.	.	.	6,555.3
70	3.98	2,429.7	4,153.5	418.0	37.1	22.8	234.0	7,295.0
77	3.86	2,329.6	4,982.9	226.9	133.9	65.0	535.6	8,273.9
84	3.85	2,302.3	5,635.5	158.6	468.7	96.2	969.2	9,630.4
91	3.69	2,191.8	4,961.5	152.8	1,120.0	122.2	1,253.9	9,802.0
104	3.47	2,103.4	4,828.9	146.3	3,396.9	65.7	1,494.4	12,035.4
111	3.86	2,374.5	6,926.4	169.7	6,019.7	252.9	1,996.8	17,739.8
118	3.59	2,589.6	6,630.7	153.4	8,457.2	85.2	2,148.9	20,064.9
127	3.79	2,810.6	6,496.1	145.6	10,030.2	55.3	2,217.2	21,754.9
139	2.90	2,555.8	6,479.9	152.8	11,659.1	152.8	2,069.0	23,069.2

In relation to leaf area index (LAI, m^2^ m^−2^), at the start of the crop cycle (day 14, V_2_ growth stage), its value was 0.06 m^2^ m^−2^ and at 70 days (R_1_ growth stage), it expanded to 3.98 m^2^ m^−2^. After this, at flowering, the leaf area index continued practically constant until 127 days (R_5_/R_6_ growth stage), with a significant drop at 139 days (R_6_ growth stage) presenting 2.90 m^2^ m^−2^ (Table 2).

Total dry matter *D* increased until the physiologic maturity (R_6_ growth stage), at 139 days with 23,069 kg ha^−1^ (354.9 g plant^−1^) for a population of 65,000 plants ha^−1^ (Table 2). The fit of total dry matter accumulation to the growth model resulted in an R^2^ of 0.97.

The Pearson correlation coefficient value (0.985) was larger than the critical value, at 10% of significance level, for the dry matter sigmoid model of the maize crop (hybrid DKB 390 VT PRO 2) as a function of time (t, d). The empirical parameters are *k*_1_ = −5,776.147 kg ha^−1^, *k*_2_ = 30,474.954 kg ha^−1^, *k*_3_ = 151,749 days and *k*_4_ = 68.397 days.

### Micronutrient content

**Figure 3:**
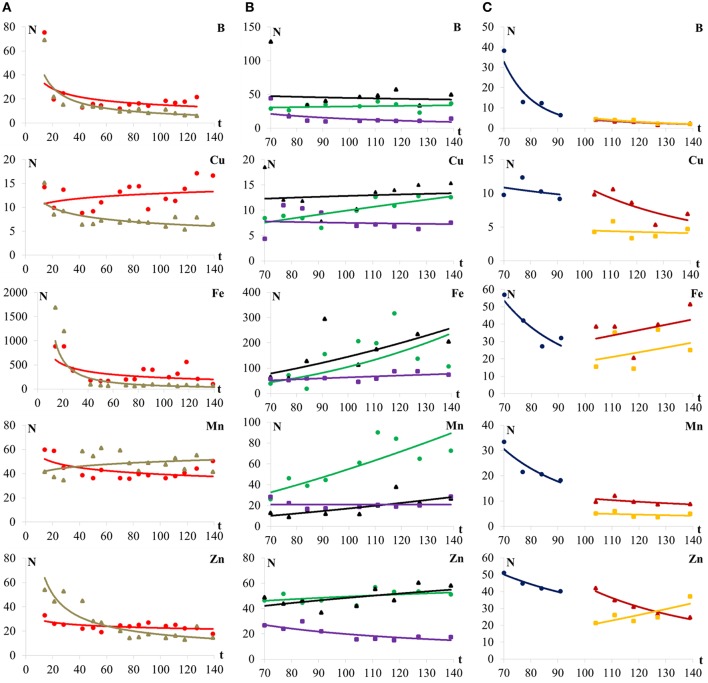
B, Cu, Fe, Mn, and Zn contents (*N*, mgkg^−1^) in **(A)** leaf (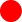), stalk (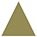), **(B)** tassel (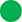), style-stigma (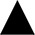), straw (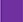), **(C)** ear (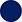), cob (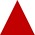), and grain (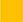) of maize plants (hybrid DKB390VTPRO2) as a function of time (t, d).

Observed micronutrient contents (B, Cu, Fe, Mn, and Zn) in leaf and stalk were initially high, decreasing to a stable value near the end of the cycle (Figure 3A). In most cases, nutrient contents in the leaf were higher than in the stalk, except for Mn, which showed a higher concentration in the stalk until day 112 (between R_4_/R_5_ and R_5_ growth stages). The concentration of Zn was slightly lower in leaves at the very early stages, but from 60 days (between V_15_ and R_1_ growth stages), its concentration in leaf and stalk decreased continuously.

In the stalk, Mn contents practically did not vary over time. Iron was the micronutrient with highest contents in the leaf and in the stalk, being higher in leaf.

For the tassel and style-stigma, in general, all micronutrients presented a concentration increase over time, except B in the style-stigma. In the straw, B, Cu, and Zn concentrations decreased over time, and Fe and Mn increased over time (Figure 3B).

The Mn content in straw was constant. Concentrations of Cu, Fe, Mn, and Zn in the tassel were higher during the final growth stages of the crop. B followed by Cu are the nutrients that present highest contents in the style-stigma. Furthermore, when comparing the three organs, one can observe greater contents of B and Cu in the style-stigma and of Fe and Mn in the tassel (Figure 3B).

In relation to the tassel, the micronutrient contents are lower at beginning and increase with crop development, with highest levels between days 111 and 118 (between R_4_/R_5_ and R_5_ growth stages).

In relation to the straw, the Fe and Mn contents are constant during the time of collection and, the Zn contents are lower in the final development growth stages of maize plant (Figure 3B). When comparing the three organs, higher micronutrient contents in the style-stigma and tassel in comparison to straw are observed (Figure 3B).

From 104 days on (R_4_ growth stage), it was observed that the micronutrient contents are higher in the ear when compared to contents in the grain (Figure 3C).

The mineral elements in the ear (cob + grain) were analyzed from 70 (R_1_ growth stage) to 91 days (R_3_ growth stage) (Figure 3C).

At 104 days (R_4_ growth stage), the analysis was made in separate for the cob and the grain (Figure 3C). The content of micronutrients is higher in the early ear development and decreases until the end of the evaluation period, 91 days (R_3_ growth stage).

As in the case of the ear, the levels of micronutrients in the cobs and grain decrease toward the end of the crop cycle (Figure 3C). However, the Fe and Mn levels are constant in cob and the levels of Fe and Zn are also fairly constant for the grain.

After the R_4_ growth stage, at 104 days, the Fe contents were higher in the grain when compared to the cob (Figure 3C).

At the R_1_ growth stage (70 days), higher B, Fe, and Mn contents were found in the ear.

In grain, there are higher levels of B, Cu, and Mn at 104 (R_4_ growth stage) and Fe and Zn at 140 days (R_6_ growth stage) (Figure 3C).

In 31 out of 45 cases, the Pearson correlation coefficient values were larger than the critical values (69%) (leaf: 80%, stalk: 80%, tassel: 60%, style-stigma: 60%, straw: 60%, ear: 80%, cob: 60%, grain: 40%, and plant: 100%), at 10% of significance level, for micronutrient content power model (Table 3).

**Table 3:** Empirical parameter values (k_5_, mgkg^−1^d ^−*k*_6_^ and k_6_) and Pearson correlation coefficient (r) for micronutrient (m) content (*N*, mg kg^−1^) power model, for B, Cu, Fe, Mn, and Zn in leaf, stalk, tassel, style-stigma, straw, ear, cob, grain, and above ground maize plant (hybrid DKB390VTPRO2).

**m**	**Leaf**	**Stalk**	**Tassel**	**Style-stigma**	**Straw**	**Ear**	**Cob**	**Grain**	**Plant**
**EMPIRICAL PARAMETER VALUES (k_5_, mgkg^−1^d ^−*k*_6_^)**									
B	93.693	336.03	16.258	95.93	3,593.6	1.0E+13	336,647	4.0E+06	4,828.994
Cu	8.408	21.183	0.2974	7.2660	12.275	55.104	63,469	17.101	0.251
Fe	2,213.2	38,302	0.0535	0.0015	4.1593	2.0E+06	0.2858	0.0323	112,537.98
Mn	75,824	33,114	0.0631	0.0183	19.722	292,534	353.59	103.96	71.34
Zn	38,335	370.12	20.272	8.2008	1,083.9	2,431.2	206,959	0.0153	111.305
**EMPIRICAL PARAMETER VALUES (k_6_)**									
B	−0.395	−0.807	0.1481	−0.165	−1.209	−6.254	−2.45	−2.959	−1.4147
Cu	0.093	−0.253	0.7613	0.1234	−0.107	−0.382	−1.877	−0.29	−0.2397
Fe	−0.489	−1.383	1.7164	2.4275	0.5916	−2.514	1.0133	1.3793	−1.5903
Mn	−0.142	0.089	1.4705	1.4864	0.0127	−2.158	−0.751	−0.65	−0.1207
Zn	−0.115	−0.666	0.7936	0.3853	−0.868	−0.913	−1.84	1.5549	−0.3687
**PEARSON CORRELATION COEFFICIENT (r)**									
B	0.604^*^	0.914^*^	0.203	0.075	0.602^*^	0.950^*^	0.785^*^	0.917^*^	0.869^*^
Cu	0.303	0.728^*^	0.771^*^	0.115	0.087	0.338	0.766^*^	0.149	0.741^*^
Fe	0.538^*^	0.876^*^	0.715^*^	0.629^*^	0.626^*^	0.877^*^	0.341	0.355	0.965^*^
Mn	0.582^*^	0.353	0.860^*^	0.762^*^	0.014	0.921^*^	0.633	0.360	0.742^*^
Zn	0.554^*^	0.894^*^	0.464	0.561^*^	0.808^*^	0.978^*^	0.979^*^	0.811^*^	0.945^*^

* *Significant at level of 10% for micronutrient content power model by the critical Pearson correlation coefficient test*.

### Micronutrient absorption *A* and absorption rate λ for the above ground plant

The total micronutrient absorption increased with crop growth and development. At 14 days, the Cu, Fe, Mn, and Zn absorptions were, respectively, 3, 430, 10, and 10 g ha^−1^, and at the end of the cycle at 139 days, 180, 1,040, 930 and 430 g ha^−1^. The absorption rate was lower at the beginning of the cycle, 14 days (V_2_ growth stage) it was, respectively for Cu, Fe, Mn, and Zn, 1, 20, 3, and 3 g ha^−1^ d^−1^, the absorption peak was observed at 111 days (R_4_/R_5_ growth stage) with a value of 2, 90, 12, and 5 g ha^−1^ d^−1^ (Table 4).

**Table 4:** Mean values of nutrient content (*N*, mgkg^−1^), absorption (*A*, gha^−1^) and absorption rate (λ, gha^−1^d^−1^) of B, Cu, Fe, Mn and Zn in maize crop (hybrid DKB390VTPRO2) in relation to time after sowing (t, d).

**t**	**Nutrient content (*****N*****)**					**Absorption (*****A*****)**					**Absorption rate (*****λ*****)**				
	**B**	**Cu**	**Fe**	**Mn**	**Zn**	**B**	**Cu**	**Fe**	**Mn**	**Zn**	**B**	**Cu**	**Fe**	**Mn**	**Zn**
14	–	13	1,693	52	42	–	3	430	10	10	–	1	20	3	3
21	65	12	888	49	36	50	10	690	40	30	2	1	20	3	2
28	43	11	562	48	33	60	20	760	60	40	2	1	30	4	2
42	24	10	295	45	28	70	30	810	120	80	2	1	30	4	2
50	19	10	224	44	26	70	40	830	170	100	3	1	40	5	3
56	16	10	187	44	25	70	40	840	200	110	3	1	40	6	3
70	12	9	131	43	23	80	60	890	290	160	4	1	50	7	3
77	10	9	113	42	22	80	70	910	340	180	4	2	60	8	4
84	9	9	98	42	22	90	80	940	400	210	4	2	70	9	4
91	8	9	86	41	21	90	100	970	470	240	5	2	70	10	4
104	7	8	70	41	20	100	120	1,030	600	300	6	2	80	11	5
111	6	8	63	40	20	100	140	1,050	680	330	6	2	90	12	5
118	6	8	57	40	19	110	150	1,070	750	360	6	2	90	11	4
127	5	8	51	40	19	110	170	1,080	840	400	5	2	80	10	4
139	4	8	44	39	18	110	180	1,040	930	430	3	1	50	6	2

**Figure 4:**
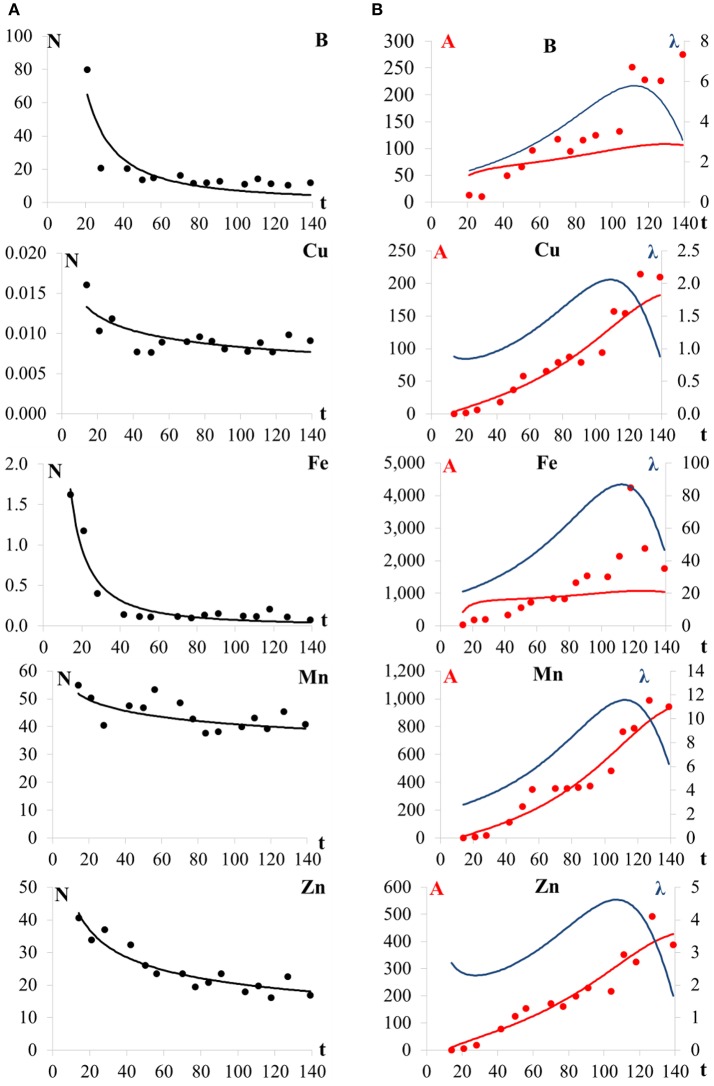
**(A)** micronutrient contents of the above ground maize plant (*N*, mgkg^−1^), and **(B)** total absorption (*A*, gha^−1^) and absorption rate (λ, gha^−1^d^−1^) for B, Cu, Fe, Mn, and Zn in the maize (hybrid DKB390VTPRO2) crop (weighted average of all above ground organs) as a function of the number of days after sowing (t, d).

Equation (1) fitted well for all micronutrients except Cu (Figure 4A). The absorption rate increased for all nutrients from 14 to 111 days. *N* is described as a decreasing power function for all micronutrients, resulting in absorption rate functions whose shape differs from the sigmoid function *D*.

The total B uptake at the R_1_ growth stage (70 days) was 80 g ha^−1^ and at the end of the crop cycle, R_6_ growth stage (139 days) 110 g ha^−1^. The B absorption rate presents a maximum value λ_max_ on 112 days (R_4_/R_5_ growth stage) (Figure 4A) with 6 g ha^−1^ d^−1^. Thereafter the rate decreases to the end of the crop cycle.

Cu had a similar behavior to Mn and Zn, but with a slightly worse fit. Nevertheless, the power model for Cu content was chosen, in the same way as for the contents of Mn. The total absorption of Cu also increased during the crop development. At day 14, the absorption of Cu was 3 g ha^−1^, at the end of the vegetative growth stage (day 56, V_15_ growth stage), the absorption was 40 g ha^−1^ and at the end of the cycle, it increased to 180 g ha^−1^. With respect to the absorption rate, the behavior of Cu was similar to Zn, being high at the first sampling (14 days), decreasing until 38 days (V_8_ growth stage) and then increasing up to the maximum value at 109 days (between R_4_ and R_4_/R_5_ growth stages) of 2 g ha^−1^ d^−1^ (Figure 4A). Thereafter it decreases continuously until the end of the crop cycle.

The total absorption of Fe increased throughout the development of the crop, at 14 days (V_2_ growth stage) showing a value of 430 g ha^−1^. At R_1_ growth stage (70 days), the Fe uptake was 890 g ha^−1^ and at the end of the cycle 1,040 g ha^−1^. With respect to the absorption rate of Fe, it was high initially, at the V_2_ growth stage (14 days) with a value of 20 g ha^−1^ d^−1^. The nutrient absorption rate of Fe increased to the maximum value of 90 g ha^−1^ d^−1^ at 112 days. The Fe rate decreases thereafter to the end of the cycle (Figure 4B).

Mn behaved similarly to Cu. The total absorption increased during crop development from 290 g ha^−1^ at the R_1_ growth stage (70 days) to 930 g ha^−1^ at the end of the cycle (R_6_ growth stage, 139 days). At 14 days, the absorption rate was low, increasing thereafter continuously until 112 days. From this period, increasing values were observed, and the maximum absorption rate (λ_max_) occurred at 112 days (between R_4_/R_5_ and R_5_ growth stages) (Figure 4B), with a value of 12 g ha^−1^ d^−1^. Then, the Mn absorption rate decreased until harvest.

Zn total absorption was equal to10 g ha^−1^ at 14 days and 430 g ha^−1^at the end of the cycle. The absorption rate peak was 5 g ha^−1^ d^−1^ at 107 days (between R_4_ and R_4_/R_5_ growth stages) (Figure 4A), thereafter decreasing until harvest.

The Pearson correlation coefficient values (0.869, 0.741, 0.965, 0.742, and 0.945) (Table 4) for micronutrient (B, Cu, Fe, Mn, and Zn) content model (Figure 4A) were larger than the critical value, at the 10% of significance level.

### Micronutrient content in stalk sap

The values for B, Cu, Fe, Mn, and Zn contents (*N*), total absorption (*A*) and the maximum absorption rates for each micronutrient (λ_max_), and maximum concentrations (C_*C*_) are shown in Table 5.

**Table 5:** Maximum concentration (*C*_*C*_, mgL^−1^ or Cc*, μM, as function of the molecular weight *M*, gmol^−1^) of each micronutrient (m) in crude sap (xylem) of maize crop (hybrid DKB390VTPRO2) at t days after sowing, corresponding to the growth stage (*GS*), above ground plant content (*N*, mgkg^−1^), nutrient absorption (*A*, gha^−1^), maximum absorption rate (λ_max_, gha^−1^d^−1^), actual evapotranspiration (*ETa*, mmd^−1^), evaporation (*E*, mmd^−1^), absorbed water by the plant to form dry matter, the water retained by the plant (α, kg m^−2^ d^−1^), total dry matter stored on the day of calculation (Δ*D/*Δ*t*, kgm^−2^d^−1^) and relative difference (ΔC, %) of *Cc* values estimated by equations 12 (Cc_12_) and 14 (Cc_14_).

**m**	***M* g mol^−1^**	***t* das**	***N* mg kg^−1^**	***A* g ha^−1^**	***λ_max_* g ha^−1^ d^−1^**	***ETa* mm d^−1^**	***E*^a^ mm d^−1^**	**Δ^b^ kg m^−2^ d^−1^**
B	11	112	5.8	103.6	6.1	1.2	0.060	0.1717
Cu	64	109	8.1	131.5	2.1	1.5	0.075	0.1711
Fe	56	112	62	1,054.6	86.8	1.2	0.060	0.1717
Mn	55	112	40.4	686.4	11.6	1.2	0.060	0.1717
Zn	65	107	19.9	309.3	4.6	1.5	0.075	0.1699
**m**	***GS***	**t das**	***ΔD/Δt* kg ha^−1^ d^−1^**	***Cc*_12_ mg L^−1^**	*Cc*_12_* μM	***Cc*_14_ mg L^−1^**	*Cc*_14_* μM	***ΔC*^3^ %**
B	R_4_/R_5_	112	289	0.47	42.3	0.51	46.2	−9.31
Cu	R_4_/R_5_	109	288	0.13	2.1	0.14	2.2	−6.40
Fe	R_4_/R_5_	112	289	6.62	118.2	7.23	129.2	−9.31
Mn	R_4_/R_5_	112	289	0.88	16.1	0.97	17.6	−9.31
Zn	R_4_/R_5_	107	286	0.29	4.4	0.31	4.7	−6.33

a *E = 0.05ETa*.

b *u = 0.9 kg kg^-1^ (plant water content) and T_H_ = 0.06 kg kg^-1^ (hydrogen content in the dry matter of maize crop)*.

*^c^ΔC = 100(Cc_12_-Cc_14_)/Cc_14_*.

The maximum absorption rate (λ_max_, g ha^−1^ d^−1^) of the micronutrients occurred between 107 and 112 days, i.e., during the R_4_ (50% of the plants exh0ibiting farinaceous grains) and R_5_ (50% of the plants exhibiting hard farinaceous grains) growth stages (Ritchie et al., 1996). These growth stages are therefore the most important with respect to the nutritional needs of the maize crop. At these growth stages, the starch accumulation in the maize grain increases featuring a period of grain filling, resulting in greater dry mass of grain.

The ETa values in the corresponding days of λ_max_ are relatively small because the period coincides with the local winter season, due to cloudy days. Estimates of the maximum concentration (*C*_*C*_, mg L^−1^) in the gross plant sap were highest for Fe, followed by Mn (Table 5).

## Discussion

### Evapotranspiration

Water absorption refers to the sum of the transpiration, the amount of water retained by the maize crop and the amount of water required to produce the total dry matter. Maize crop productivity depends fundamentally on the water absorption and the carbon dioxide assimilation, since carbon and oxygen (from CO_2_), and hydrogen (from H_2_O) represent about 96% of the dry matter.

The maize crop potential evapotranspiration (water requirement) during the whole cycle was 180 mm. The total actual evapotranspiration was 135 mm. There was a water deficit of 45 mm. A mild water stress occurred during 38 days of crop establishment, from April 14 to May 21.

### Water deficit

Effects of water deficit vary according to the development growth stage of the crop, and when occurring in the vegetative period, plant height was reduced, leaves become smaller, and consequently present a lower leaf area. However, the stress is mainly harmful to grain productivity when it occurs at the beginning of the tassel development up to grain filling. The demand for water increases rapidly about 2 weeks before the development of the tassel, and reaches a maximum at the flowering peak, continuing high for about two more weeks when it decreases rapidly.

### Crop cycle

The cropping period was from March to August, a season that is called “second maize harvest” in Brazil, nowadays more important than the “main maize harvest,” from September to March. The crop cycle had a duration of 139 days. The vegetative phase ended 70 days (R_1_ growth stage), accumulating 863°C days, and the reproductive phase extended from 70 to 139 days (R_6_ growth stage), with a total of 733°C days (Table 1).

### Crop growth and development

At the end of the vegetative phase, the maize plant gives priority to the development of the tassel and ear, which causes a remobilization of the photo-assimilates and nutrients from the leaf to the production and dispersion of pollen and to grain filling. It is also possible to observe a slight reduction of stalk dry matter during 91–104 days (R_3_ and R_4_ growth stages) (Table 2).

During the reproductive phase, the ear is a significant physiological drain. The plant redistributes photo-assimilates and nutrients to the grain, for filling. Therefore, the closer to physiologic maturity, the more leaves and stalk enter in senescence.

The accumulation and mobilization of dry matter in maize crop has a characteristic sequence over the growth cycle. For the maize crop, some authors report senescence as a process that encompasses the above ground plant, besides being caused by internal and external factors and mediated by a genetic program.

At 112 days, the values of the growth rate continue to be positive (dY/dt), but with decreasing daily gains (d^2^Y/dt^2^), i.e., the maize plant reduces its rate of dry matter accumulation because of the senescence process.

### Micronutrient content

The demand for micronutrients depends mainly on the crop productivity (production of total dry matter mass per unit area) (Bray, 1948), and the variation of dry matter composition of genotypes of the species of interest (least significant component).

Studies of Ciampitti et al. (2013), evaluating the contents of B, Cu, Fe, Mn, and Zn in maize plants, showed similar results as those found in this study. These micronutrient contents are larger at the beginning of the development of the maize crop, and then decrease up to physiological maturity.

During the initial growth, as there is a low production of plant dry matter, a high concentration of micronutrients was found coming from the soil or being remobilized from other parts of the plant.

The absorption of micronutrients depends on the water absorption and the effective content of the micronutrient in the soil solution. Transpiration depends on the difference between water potential in the leaf and in the atmosphere. The absorption of water and nutrients depends on the elements of the climate (such as temperature, relative humidity and wind speed), crop (such as root system architecture, leaf area index and productivity) and soil (water and micronutrients content).

With crop growth, which usually follows a sigmoidal model, the dry matter accumulation is more expressive than the capacity of the plant to absorb and concentrate micronutrients. Therefore, we have a dilution effect due to the maize crop growth. Furthermore, it is known that higher concentrations of Fe and B, for example, are related to leaf and stalk. Over time, other structures gain greater proportion in the share of total dry matter thus contributing to part of this dilution effect.

### Micronutrient content in stalk sap

Due to complex factors related to the interaction between genotype and environment (climate and soil), the determination of the critical content in the xylem solution is the proposed procedure to evaluate soil fertility. Among the different factors, we highlight: phenological stage, effective depth of the root system corresponding to at least 90% of the potential evapotranspiration of the crop, distribution of the root system in the soil profile, soil water flow density, root trapping, mass flow and diffusion of micronutrients in soil, pH, temperature and content of the different micronutrients in soil solution and evapotranspiration, mainly.

During photosynthesis, the produced carbohydrate (CH_2_O) molecules are composed of carbon (C) and oxygen (O) atoms from atmospheric CO_2_, whereas the hydrogen (H) originates from water molecules from the soil. The produced O_2_ returning to the atmosphere also originates from the soil water molecule (Taiz and Zeiger, 2006):

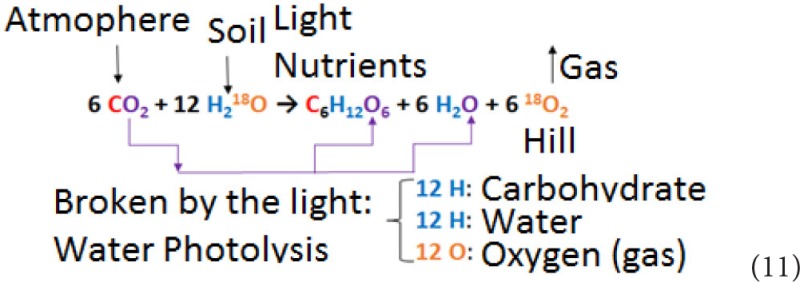



This reaction is endothermic, requiring energy that, in the photosynthesis process, is provided by solar radiation, where (i) 6 carbon and 6 oxygen atoms of the produced carbohydrate molecule are derived from the enzymatic breakdown of atmospheric carbon dioxide by RuBisCO (Ribulose-1,5-bisphosphate carboxylase/oxygenase); (ii) 12 atoms of hydrogen of the produced carbohydrate molecule are derived from the extracted water from the soil and broken by the light in the leaf, process known as water photolysis; (iii) the other 12 atoms of hydrogen with 6 atoms of oxygen will build 6 molecules of water; and (iv) the 12 atoms of oxygen of the molecule of oxygen (O_2_), that return as gas to the atmosphere, are derived from water molecule: reaction proven by Robin Hill in the years 1960 using labeled oxygen (^18^O). Then, the absorbed water responsible for the total dry matter produced during the day of calculation can be estimated as $9T_{H}\frac{dD}{dt}$ and the water retained by the plant can be estimated as $9T_{H}\frac{dD}{dt}/\left(1-u\right)$, so that the maximum concentration of a micronutrient in the stalk sap (Cc, mg L^−1^) is:

(12) $$\begin{array}{r} Cc=\frac{\lambda_{\max}}{10^{4}\left[ETa-E+9\left(\frac{2-u}{1-u}\right)T_{H}\frac{dD}{dt}\right]} \end{array}$$

where *ETa* is the actual evapotranspiration (mm d^−1^), here calculated using the simple Thornthwaite and Mather (1955) water balance, *E* the soil surface evaporation (mm d^−1^), *u* the plant water content (kg kg^−1^), *T*_*H*_ the hydrogen content in the dry matter of maize crop (0.06 kg kg^−1^) and d*D/dt* the total dry matter stored on the day of calculation (kg m^−2^ d^−1^):

(13) $$\begin{array}{r} \frac{dD}{dt}=\frac{-2k_{2}{k_{4}}^{2}\left(t-k_{3}\right)}{{\left[{k_{4}}^{2}+{\left(t-k_{3}\right)}^{2}\right]}^{2}} \end{array}$$

*Cc* represents the critical concentration λs (mg L^−1^) in the soil solution. In this way, knowing *Cc*, it should be possible to develop a methodology for characterizing soil fertility and to recommend fertilization optimized to reach the maximum productivity as a function of the limiting nutrient.

With the aim of estimating the micronutrient maximum concentrations (Cc, mg L^−1^) in the maize stalk sap at the time of maximum absorption, Equation (12) was simplified to:

(14) $$\begin{array}{r} Cc=\frac{\lambda_{\max}}{10^{4}.ETa} \end{array}$$

The elimination of the term [$-E+9\left(\frac{2-u}{1-u}\right)T_{H}\frac{dD}{dt}$] results in an underestimation of 6.33% (Zn) to 9.31% (B, Fe, and Mn) (Table 5).

Experiments carried out with the aim of evaluating the absorption of nutrients by a maize crop also report that the increased absorption of nutrients B, Cu, Fe, Mn, and Zn occurred during reproductive phase, between R_4_ and R_5_ growth stages (Ciampitti et al., 2013).

The maximum concentration (C_*C*_, mg L^−1^) of each nutrient in the xylem sap is here assumed to be related to the soil solution absorbed by plant roots (Table 4). Based on the analysis of the results obtained in this research, it is suggested that future studies should be conducted in more than one growing season, with replicates of several years or even at different times. Such experiments may include different genotypes, as well as different regions, varying the population of plants in the experimental area, and simulate high, medium and low technology managements.

It is not possible to separate the effect of lack of water from the lack of micronutrients in the loss of maize crop productivity. The critical micronutrient content (*Cc*) is related to the transpiration and productivity (related to the maximum maize crop demand λ_max_). Theoretically, the micronutrient with lowest content in the stalk sap (related to the lowest soil offer λ*s*) defines productivity. On the other hand, the limiting maximum micronutrient absorption rate (λ_max_) corresponding to the lower productivity defines the attainable productivity limited by water and micronutrient (Liebig's minimum law).

### Water and nutrients absorption by plants

In nature, water and nutrient absorption occurs simultaneously (with and without energy expenditure), because the solute movement in the soil occurs by the combined processes of diffusion and mass flow, which in both cases are related to dynamics of water in the natural system composed by soil, plant and atmosphere phases.

From the thermodynamic point of view, the soil chemical fertility depends on the physical process related to the water movement in nature. The transpiration depends, among other factors, on the water potential difference between vapor in the atmosphere and liquid water in the leaf.

The water potential (ψ, atm) in the atmosphere phase defines the magnitude order of transpiration, which depends on air temperature (T, K) and relative humidity (Ω, kPa kPa^−1^):

(15) $$\begin{array}{r} \text{Ψ}=55.5.R.T.\ln\;\left(\Omega \right) \end{array}$$

which depend on other climate elements such as solar radiation, rainfall and wind, for example.

### Maximum micronutrient concentration in the sap, productivity and transpiration

The water absorption by a crop is given by the sum of transpiration and constitutional water dependent on dry matter production. Absorption of nutrients is dependent on water absorption (or transpiration if we neglect constitutional water) and nutrient concentration on the absorbed solution. Therefore, high nutrient concentration in the soil solution (higher chemical fertility in the current classical model, since it does not reach salinization levels) is correlated with high concentration in the plant xylem solution, greater nutrient absorption, high dry matter production (of the different organs) and greater productivity (yield).

Therefore, this approach (preliminary studies to characterize the temporal variation of micronutrient composition of the above ground organs of maize and correlated uptake rates) allows developing a new concept to change the classic criteria of fertilization recommendation taking into account the reality of the facts (high dependence of micronutrients absorption to water absorption). The experimental data are used merely to give an example of application and to show the order of magnitude of the micronutrients contents in different organs and the micronutrients concentrations in the sap.

**Figure 5:**
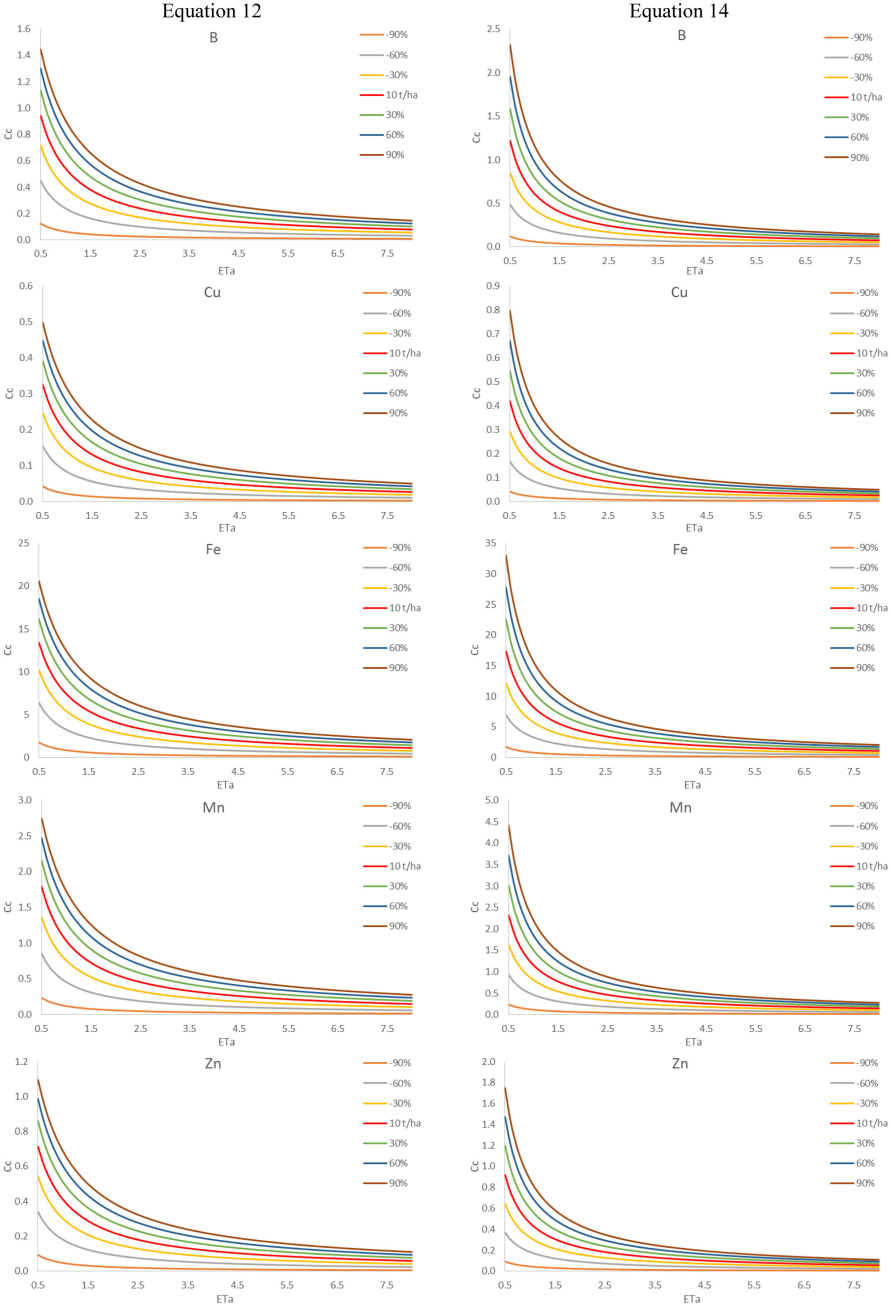
B, Cu, Fe, Mn, and Zn maximum concentrations (Cc, mgL^−1^) in the maize stalk sap estimated by complete (Equation 12) and simplified (Equation 14) equations for the maize crop as a function of yield (lower and higher than 10tha^−1^) and actual evapotranspiration (ETa, mmd^−1^).

Figure 5 shows the micronutrient (B, Cu, Fe, Mn, and Zn) maximum concentrations in the maize stalk sap at the time t_max_ estimated by Equations (12) and (14) for the maize crop as a function of low (lower than 10,000 kg ha^−1^) and high (higher than 10,000 kg ha^−1^) yield and actual evapotranspiration. It is observed that the maximum concentration Cc presents high variation as a function of productivity in an environment of low evapotranspiration, which does not occur under high evapotranspiration. It was also observed that the simplified equation (Equation 14) presents smaller errors under the condition of high evapotranspiration.

### Final considerations

This preliminary study may serve as a basis for other researchers to develop an alternative methodology to the current procedure using chemical soil extractors. This alternative may vary from the measurement of the micronutrient content in the xylem in the indicator plant, which may be the species of interest at the point of maximum growth rate as described in this work, or even through the correlation of this value with the value measured in seedlings of the same species or using a native species present in the area (Cate and Nelson, 1965).

Chlorine, molybdenum and nickel were not considered in the present study due to the low amounts of these micronutrients used in maize fertilization programs, in general, for the following reasons: (i) chlorine is supplied when potassium is applied as KCl (about 40 kg ha^−1^ of K_2_O), (ii) molybdenum is most frequently used in soybean cultivation due to the symbiosis with *Bradyrhizobium* sp. and (iii) nickel presents few research results because its essentiality was found only recently.

The next steps of this preliminary study would be including roots and validating the model under different environmental conditions and with different genotypes. In this way, the model is able to take into account the effect of the environment and of the genotype that were not considered in this initial work, as well as to characterize the chlorine, molybdenum and nickel contents of different organs and their respective extractions.

## Conclusions

We proposed a methodology that can be used for characterizing the micronutrient absorption rate of crops and tested it for a maize growing scenario. Results show that: (i) the micronutrient content variation in time follows a power model, with higher values for the initial growth stages of development and leveling off to minimum values at the R_6_ growth stage, (ii) the maximum micronutrient absorption rates occur in the reproductive growth stages, and (iii) these evaluations allowed to predict the maximum need for micronutrients, considered equal to their concentration in the stalk sap. The proposed methodology can be used as a basis for further improvement in micronutrient fertilization of maize and other crops.

## Author contributions

KM and DD designed the research; JF, FS, GF, and SM performed the research; KM, DD, KR, and Qd analyzed data; KM, DD, KR, and Qd wrote the manuscript.

### Conflict of interest statement

The authors declare that the research was conducted in the absence of any commercial or financial relationships that could be construed as a potential conflict of interest. The reviewer YZ and handling Editor declared their shared affiliation, and the handling Editor states that the process nevertheless met the standards of a fair and objective review.
